# Using an academic-community partnership model to deliver evidence-based falls prevention programs in a metropolitan setting: A community case study

**DOI:** 10.3389/fpubh.2023.1073520

**Published:** 2023-03-30

**Authors:** Cathy S. Elrod, Sara T. Pappa, Patricia C. Heyn, Rita A. Wong

**Affiliations:** Center for Optimal Aging, Marymount University, Arlington, VA, United States

**Keywords:** older adults, evidence-based programs, fall prevention, academic-community partnership, implementation

## Abstract

**Background:**

Prevention is an effective approach for mitigating the negative health outcomes associated with falls in older adults. The Administration for Community Living (ACL) has sponsored the implementation of evidence-based falls prevention programs (EBFPPs) across the United States through cooperative agreement grants to decrease the health and economic burden of falls. Marymount University received two of these grants to deliver three EBFPPs into the northern Virginia region. This community case study describes the development of a collaboration between a university and community-based organizations to adopt and implement multiple evidence-based programming in an area where none previously existed.

**Methods:**

Through an academic-community partnership, EBFPPs were introduced to and implemented by senior-focused organizations. Target adopters were senior and community centers, multi-purpose senior services organizations, recreational organizations, and residential facilities serving older adults. The three EBFPPs were (1) Stay Active and Independent for Life (SAIL), (2) a Matter of Balance (MOB) and (3) Otago Exercise Program (OEP). Key interdependent project elements included: (1) fostering ongoing community organization collaboration, (2) introducing programs in the community, (3) growing and sustaining delivery sites, (4) preparing trained program leaders, and (5) building community demand for the programs.

**Results:**

From August 2016–June 2022, 5,857 older adults participated in one of the three EBFPPs. SAIL classes were offered at 33 sites and MOB workshops at 31 with over 70% of them occurring at community or senior centers. OEP was offered at 4 sites. Factors that influenced the implementation of these programs included having: key advocates at host organizations, programs embedded into site workflows, sufficient capacity and workforce, engaged invested partners, and flexibility in working with a complex set of agencies and systems with different administrative structures.

**Conclusion:**

By connecting academic faculty with various community members from multiple sectors, new initiatives can be successfully implemented. Results from this ACL-funded project indicate that using an academic-community partnership model to build relationships and capacity for ongoing delivery of health promotion programming for older adults is feasible and effective in delivering EBFPPs. In addition, academic-community partnerships can develop a strong network of invested partners to foster continued support of fall prevention activities.

## Introduction

Falls in older adults are a public health problem as more than 25% of older adults fall each year (1, 2). Although many falls go unreported, they frequently lead to injuries and are associated with substantial health care costs. In 2015, Medicare spent an estimated $28.9 billion for medical costs associated with nonfatal falls in older adults (3). In addition to the direct medical costs associated with falls and fall-related injuries, falls are known to be associated with long-term negative health effects including disability and increased isolation from fear of falling with self-imposed limits on community activity (1, 4, 5). Falls may also contribute to a reduced quality of life.

Risk factors for falls are well-established (6–8) and prevention is an effective approach for mitigating the negative health outcomes associated with falls (9). Evidence-based and community-delivered fall prevention programs that increase awareness of risk factors and engage older adults in falls-prevention targeted exercise and educational activities have been successfully administered in group settings (10–12) and shown to effectively reduce falls, fear of falling, and health care costs (11–13). However, implementing these proven community programs in diverse and varied “real world” settings can be challenging (14–16).

In 2016, community advocates from local senior serving organizations and agencies identified a lack of falls prevention programs in the region as a significant unmet service. Although various community groups hosted a range of general fitness classes, none of the classes were identified as ones that address fall prevention, and there was no public or private senior-serving regional agency to coordinate outreach, implementation, and training across the various jurisdictions. Marymount University (MU) was known for strong community ties across the region and had a history of providing fall prevention lectures and screenings. When the community advocates identified a grant opportunity to build a regional network to offer fall prevention programs, they turned to MU for partnership. MU faculty collaborated with them to write an application for a grant from the Administration for Community Living (ACL).

Since 2014, the ACL has provided funding opportunities aimed at helping communities across the United States implement and sustain community-delivered evidence-based falls prevention programs (EBFPPs) (13). MU received two cooperative agreement grants from the ACL for a project to promote the implementation and sustained delivery of three different EBFPPs in the Northern Virginia region.

Since partnering with communities is a key aspect of health promotion, this community case study describes the development of a collaboration between a university and local community-based organizations to adopt and implement multiple evidence-based fall prevention programs, each one targeting older adults at different levels of fall risk, in a large metropolitan setting. This academic-community partnership serves as a model for ways in which organizations with similar goals can work together to successfully deliver evidence-based programming in an area where none previously existed.

## Context

### Setting

The initial project, in 2016, included four jurisdictions within Northern Virginia (NoVA): Arlington County, Fairfax County, Loudoun County and the City of Alexandria. These jurisdictions, in the aggregate, are home to nearly 200,000 individuals 65 years of age or older (17). The region is broadly diverse in age, race, ethnicity, economic status, health status, culture, and language (18). The second grant project, in 2018, added the neighboring jurisdictions of Prince William County in NoVA, Montgomery County in Maryland, and the District of Columbia to the target region. Given this expansion, the estimated number of individuals in the target area who were 65 years of age or older increased to over 250,000. These regions were chosen because of their proximity to the university and the need for services identified by community leaders and advocates.

The target population was community-dwelling older adults aged 65 and older who were interested in staying active and minimizing their risk of falling. Community organizations targeted as sites for programs were primarily senior and community centers, multi-purpose senior services organizations, recreational organizations, and senior-focused residential facilities.

### Programs offered

#### Evidence-based falls prevention programs

Although falls are more likely to occur in individuals who are frail, the risk of falling increases with age regardless of one’s functional status (19). Thus, three EBFPPs, Stay Active and Independent for Life, A Matter of Balance, and the Otago Exercise Program, were chosen to provide interventions to older adults across a wide range of functional abilities and falls risk. Together these three programs with options for individuals at low, moderate, or high risk of falling have broad reach and applicability.

##### Stay Active and Independent for Life

Stay Active and Independent for Life (SAIL) is a strength, balance, and fitness program for older adults who are at low to moderate risk for falling. It consists of an hour-long exercise class that meets at least twice weekly and is led by a certified lay leader. The classes are typically provided in 12-week sessions but intended for participants to continue across multiple sessions. Participation in this multi-component exercise and education program leads to improved strength, balance and performance of daily activities in community-dwelling older adults (20, 21).

##### Matter of Balance

A Matter of Balance (MOB) includes eight 2-hour small group sessions led by two trained leaders. The program uses a cognitive-behavioral approach that focuses on increasing self-efficacy (22, 23). It is designed to reduce the fear of falling and familiarize older adults with balance-focused exercises. Lay leaders are trained as MOB coaches and facilitate workshops in communities. Findings from research studies support its ability to reduce fear of falling and avoidance behaviors, and increase balance confidence (24–26).

##### Otago Exercise Program

The Otago Exercise Program (OEP) was developed for older adults at high risk of falling (27). It consists of a series of 17 strength and balance exercises with a walking component. Studies have shown it leads to improved balance, lower leg strength, physical fitness, and self-confidence (28). It was originally designed to be delivered in the home by a physical therapist (PT), but in the United States this model encountered multiple implementation challenges because of Medicare reimbursement requirements (29, 30). Newer models of delivery that include community-based group classes have comparable outcomes (31–34).

### Academic-community partnership

For this project, the roles of the academic partner, faculty within Marymount University, were to assist the community in implementing their identified needs for fall prevention programs, work collaboratively to establish an implementation and sustainability plan, train EBFPP leaders, provide fidelity checks and annual booster sessions for program leaders, offer community lectures and fall risk screenings to older adults, and maintain a communication mechanism with all partners.

The community partners’ roles were to establish community-defined needs, work collaboratively with the academic partner to identify program leaders and sites to host the EBFPPs, participate in regular communication with the academic partner, and foster the sustained delivery of EBFPPs within their sites.

The roles in the academic-community partnership described here are consistent with prior definitions and descriptions of an academic-community partnership as “the coming together of diverse interests and people to achieve a common purpose *via* interactions, information sharing, and coordination activities” (35). Drahota et al. further developed a conceptual definition during their systematic review, “Community-academic partnerships are characterized by equitable control, a cause(s) that is primarily relevant to the community of interest, and specific aims to achieve goals(s), and involves community members (representatives or agencies) that have knowledge of the cause, as well as academic researchers” (36). Academic community partnerships can maximize resources, increase capacity among collaborators and extend the reach of health-focused programming (37). Building partnerships is key to promoting evidence-based programming and identifying policies and practices that can optimize the lives of older adults (38).

## Details of project implementation

Five key interdependent project elements included: (1) fostering ongoing community organization collaboration, (2) introducing programs into the community, (3) growing and sustaining delivery sites, (4) preparing trained program leaders, and (5) building community demand for the programs. Success or failure of any one of the five elements impacted the other elements. All five elements required concurrent focus, particularly in the early years of infrastructure building.

### Fostering ongoing community organization collaboration

The 17 community partners that collaborated on the initial grant proposal became the Steering Committee for the funded project. These included representatives from each of the four Area Agencies on Aging, two hospital systems, three different Neighborhood Village groups (locally-based networks of neighbors helping neighbors age in place), one residential living community, two senior community centers, two parks and recreation groups, one adult day health center, and several volunteer community advocates. They met quarterly to guide program implementation and to establish a permanent network of community groups to enhance communication and collaboration to support fall prevention efforts. Within 1 year, the group was formalized into the Northern Virginia Falls Prevention Alliance (NVFPA), a member organization, supported by MU, whose mission is *to maximize independence and improve quality of life of older adults by reducing falls and fall-related injuries.* NVFPA is open to all senior-serving groups and individuals interested in facilitating falls prevention efforts. It also became a founding member of the statewide Virginia Arthritis and Fall Prevention Coalition.

### Introducing programs into the community

SAIL was the first program to be offered. Many potential sites already provided exercise-based fitness programs to older adults and SAIL fit well with this format. It was initially offered at two senior living communities that were key partners on the project. These sites were well positioned with an interested fitness instructor, a committed administration, space to hold group classes, in-house program marketing capabilities, and a large pool of older adults with generally high participation rates in center activities. MOB was introduced 4 months after SAIL at two different senior community centers. The first OEP program was offered in 2018, 2 years after the start of the project. One assisted living facility and one adult day health center served as the initial sites for OEP.

### Growing and sustaining delivery sites

Positive feedback about SAIL and MOB from participants and leaders influenced other organizations to request support to start a program. A part-time program marketing coordinator created a marketing campaign to ensure consistent program and project messaging. This individual, with regular input from the project team, identified and reached out to community-based organizations, initially targeting community sites with a history of implementing health promotion programs. In-person outreach visits (3–4 monthly) to potential delivery sites by project staff served to educate, answer questions, provide fall prevention written material, and problem-solve real or perceived barriers to program implementation. A database of potential and active sites was developed and regularly updated to track all communications and recruitment efforts. Meetings of the NVFPA regularly included time for sites hosting EBFPPs to provide updates, discuss successes and challenges, and solicit advice and guidance.

### Preparing trained program leaders

It was essential that a sufficient and ongoing pipeline of trained leaders to support programs was established. SAIL and MOB use a train-the-trainer, lay-leader model for program delivery. A two-step process occurred to meet this need. First, two academic faculty were trained as Master Trainers for SAIL and MOB. Once the Master Trainers were in place, training workshops to prepare leaders were held. The second step was to develop a mechanism to deliver ongoing trainings. The Regional Training Office (RTO) was created to provide and sustain an adequate leader workforce. OEP leader training occurred through an online and asynchronous training program, offered at a modest fee by another university. RTO staff coordinated OEP leader reimbursement of this fee.

#### Regional Training Office

The Regional Training Office (RTO) was housed at MU. A part-time RTO coordinator assisted with the logistics of setting up trainings, managing communications with interested individuals, and maintaining a registry of trained program leaders in the region. This individual also updated a database of programs that included site, geographic area, leader, days and times offered, start and end dates, and data collected (attendance, pre-program and post-program participant surveys). This information was also used by the coordinator to help promote programs through connections made *via* the NVFPA and postings on their website.

Within the RTO structure, Master Trainers for SAIL and MOB delivered fidelity management and workshops to booster practice along with program leader trainings. Two program updates were provided annually for SAIL and MOB. These were offered to all trained leaders at no cost. The updates included fidelity reminders, program updates or changes, and a report out of successes and challenges experienced by leaders. Services provided *via* the RTO included a training academy for MOB and SAIL, assistance with the implementation of new EBFPPs, a speaker’s bureau for community education on falls prevention, and direction for OEP certification. A small fee was charged for leader training, primarily to cover required printed materials.

Master Trainers organized day-long trainings for leaders and assisted them with the implementation of programs. Various mechanisms were utilized to recruit new lay leaders and the training was open to all. Preference was given to leaders who were recruited by a site to become trained so they could lead programs at that site. Project staff also provided short presentations at senior-focused centers, posted flyers throughout the community, and encouraged word-of-mouth sharing. The time and date of training workshops were posted on the NVFPA website and registration was completed online. In addition to seeking EBFPP leaders from the community, university faculty developed a student-focused service-learning course that included training students to serve as a program leader, thus expanding the number of leaders and encouraging intergenerational engagement.

### Building demand for programs among older adults

Project staff and the implementation site staff shared the responsibility of marketing the EBFPPs. Project staff provided lectures and fall risk screening activities for older adults, hosted informational booths at health fairs, distributed flyers, and posted announcements on senior-focused websites and newsletters. They also provided Senior Ambassadors (SA) with information about falls and fall prevention programs to share with their community. SAs are trained community volunteers who share information with older adults about community services. Sites implementing EBFPPs also had a major responsibility for marketing to their target audiences and encouraging participation. This included internal marketing of the program, identifying members who could serve as program champions to encourage participation, and advising on the best time of the day/week to hold the program. Many sites had full schedules of activities and therefore needed several months of lead time to add a new program.

## Results

All ACL EBFPP grantees were required to enter program data into a national falls data repository. Grantees were provided with data collection forms for this purpose. Program leaders were asked to voluntarily assist in the data collection by administering ACL-provided surveys to each participant and keeping attendance logs. As collection of this data was not a component of the EBFPPs but a request of the grant funding agency, participation by the leaders was encouraged but not required. Data about the type of site was collected for the organization hosting the EBFPP. The project was approved by the university’s institutional review board.

### Participants

The target population was community-dwelling older adults aged 65 and older who were interested in staying active and minimizing their risk of falling. From August 2016–June 2022, 5,857 older adults participated in one of the three EBFPPs. Table 1 displays key participant characteristics, by program, of those who completed the pre-program participant survey: 93% were 65 years of age or older (average age = 76.6); 64% had ≥2 chronic conditions. Overall, 33% of participants completed the pre-program participant survey. Completion rates varied greatly by program and during the COVID-19 pandemic vs. pre COVID-19 time periods. COVID-19 isolation precautions required all programs to unexpectedly and immediately transition from in-person to remote delivery. Pre COVID-19, 83% of MOB participants completed the pre-program survey compared to 41% during the pandemic. Similarly, 45% of SAIL participants completed the pre-program survey prior to the COVID-19 pandemic but only 4% during it.

**Table 1:** Participant characteristics.

	SAIL (*n* = 1,317)	MOB (*n* = 465)	OEP (*n* = 53)
At or over 65 years of age	92%	94%	92%
At or over 80 years of age	37%	37%	45%
Lives alone	46%	51%	20%
Seldom active/prefers sedentary activities	9%	19%	27%
2 or more chronic conditions	62%	65%	80%
4 or more chronic conditions	13%	17%	45%
Self-reported health as excellent or very good	44%	40%	35%
Self-reported health as fair or poor	12%	12%	10%
Fell in the last 3 months	13%	34%	24%
Fall resulted in injury	9%	15%	42%
Self-reported fear of falling as ‘a lot’ or ‘somewhat’	35%	52%	33%
Concerns about falling interfered with social activities (extremely, quite a bit, moderately)	22%	32%	25%
History of depression	12%	18%	30%

Additionally, during the first 4 years of the project, MU’s IRB committee required written informed consent from participants as well as the immediate availability of a project team member to answer any participant questions during the review and consent process. This negatively impacted data collection efforts. This requirement was removed in year 5, allowing program leaders to read an ACL derived falls prevention program group leader script that described how the de-identified data was to be used prior to requesting completion of the survey forms.

### Leaders

From 2016 through June 2022, 385 SAIL leaders and 141 MOB leaders were trained, and 16 individuals reimbursed for OEP certification training. For SAIL and MOB, there were 25 and 15 leader trainings held with an average attendance of 15 and 9, respectively. Trained individuals included paid staff from sites hosting programs, independent contractors and volunteers. Some sites trained multiple people to allow flexibility in scheduling and staff availability. Training was open to all and not all leaders were from the region or had specific plans to offer a program. Natural attrition occurred over time with leaders changing jobs or work responsibilities, retiring, or moving.

### Program sites

Initially, in 2016, there were 0 sites that offered EBFPPs in the targeted region. By July of 2022, 157 SAIL sessions had been offered at 33 sites, and 57 MOB workshops at 31 sites. Seventy-three percent (73%) of the SAIL sessions and 72% of the MOB workshops were held at either community or senior centers. OEP was offered at a community day health program, senior center, and residential living facilities. Table 2 provides a full list of site locations.

**Table 2:** Setting characteristics.

	SAIL		MOB		OEP	
	Sites (*N* = 33)	Programs held at sites (*N* = 157)	Sites (*N* = 31)	Programs held at sites (*N* = 57)	Sites (*N* = 4)	Programs held at sites (*N* = 5)
Community center	7	12	7	13	0	0
Nonprofit adult daycare	0	0	0	0	1	1
Educational institution	1	8	1	1	0	0
Faith-based organization	0	0	2	2	0	0
Fitness organization	1	1	1	1	0	0
Health care organization	2	3	3	8	0	0
Local neighborhood village	1	7	1	1	0	0
Multipurpose social services	1	2	1	1	0	0
Nonprofit service organization	1	8	0	0	0	0
Residential facility	4	15	6	10	2	3
Senior center	15	101	9	20	1	1

Program adoption was operationally defined as a site that hosted the same program 3 or more times during the target period of July 2016–June 2022. Given this definition, 20 of the 33 SAIL sites adopted SAIL (61%); 7 of the 31 MOB sites adopted MOB (23%); and 0 of the 4 OEP sites adopted OEP. Only 2 sites adopted both SAIL and MOB.

### Community partners

By the end of the project time period NVFPA had over 135 members. Table 3 describes the organizational characteristics of the members. To enhance communication across all community partners, the NVFPA maintained a website (www.novafallsprevention.com) where members of the community, as well as area professionals, could find updated information on current programs, services, and events; falls prevention education and awareness resources; and links to member organizations. The group distributed an electronic newsletter 3–4 times a year and led the region in annual Falls Prevention Awareness Week activities each September.

**Table 3:** NVFPA membership: types of organizations.

Type of organization	Number of organizations
Community-based nonprofit	13
Home care (Unskilled)	12
Physical therapy/occupational therapy	10
Community center	8
Individual	8
Area agency on aging	6
Academic institution	6
Assisted living community	6
Hospital system	4
Low-income housing	4
County health department	3
County parks and recreation department	3
Senior housing	3
Media	1
Nonprofit adult daycare	1
State health department	1

## Discussion

Using an academic community partnership model, academic faculty and community partners built the infrastructure for nearly 6,000 older adults to participate in one of three EBFPPs: MOB, SAIL, or OEP. SAIL was adopted at a much higher rate than MOB, and OEP was not successful in being offered with any consistency. Key lessons learned from the implementation of this project are summarized below.

1. Secure strong organizational support.

Challenges existed in convincing sites to include new programs in their weekly workflow. While senior and community centers are a natural fit for hosting these programs, issues such as staff time, startup concerns with new initiatives, space constraints, and lack of a consistent pool of trained leaders prevented many sites from adopting the programs. Organizations that used paid staff as leaders or those that had an administrator that was a champion of the initiative were more successful in offering and adopting EBFPPs. Local champions have been shown to be key for program adoption, as they can work internally to facilitate organizational support (39, 40). Having staff view leading a program as a part of their work facilitated continued delivery of programs (41). Volunteer leaders were also used by community-based organizations to fill workforce needs. Although effective, this strategy can be problematic as a long-term solution given the inconsistencies associated with volunteer leader attrition (42).

1. Provide mechanisms to show organizations the benefits of offering EBFPPs.

Data collected from the pre-program and post-program surveys were compiled by RTO staff into one-page summaries for each host organization. This helped to illustrate the impact of their particular program on the participants’ health, quality of life indicators and satisfaction with the program. The program summaries provided individualized data to show administrators that supporting the program was worthy of their time and resources. Program leaders were also encouraged to collect testimonials from participants to be used in newsletters and promotional materials.

1. Determine how building and maintaining workforce capacity will be achieved early in the implementation process.

Building and maintaining sufficient training capacity and workforce was led by the RTO. Factors that influenced the implementation of the EBFPPs included offering a sufficient number of trainings to support new programs, assuring time for Master Trainers to conduct fidelity checks in the field and mentor newly trained leaders, and determining a feasible cost structure for the trainings. Through the relationships built within the community, partner sites offered their space at no cost for the leader trainings. Because of grant funding and a desire to entice host organizations to support having their staff trained, the fees for the leader trainings were set to cover the cost of materials only. Changes in the availability of volunteer leaders and employment or job responsibilities of staff members leading programs resulted in a continual need to replenish leaders. Thus, being diligent in continuing to secure new leaders is crucial to ensure sufficient availability to meet current and new program needs (43). Not only is it necessary to have a mechanism to train leaders, it is also critical to have enough Master Trainers to lead the trainings. The Master Trainers also need to have time allotted within their workload to mentor new leaders and ensure they are maintaining fidelity to the EBFPP.

1. Develop internal spreadsheets to manage and track communications between project team members regarding outreach to community-based organizations.

As each jurisdiction in the project area functioned in different ways, it was challenging to find a streamlined mechanism to engage with all of them collectively. This complexity required multiple processes for connecting with and assisting organizations to successfully deliver EBFPPs. What worked well in one county or city did not necessarily work well in another area. This held true in marketing and advertising, recruiting participants, utilizing referral sources, and finding staff and volunteers leaders. Internal spreadsheets provided a mechanism to take notes and document individualized needs across the diverse host organizations. Templates were made for marketing materials and recruiting leaders. As the community became more aware of the EBFPPs and delivery processes, this challenge diminished.

1. Consider incentives for completion of data collection forms.

Collecting data about participants and the impact of participation in the program is a powerful tool for demonstrating effectiveness and supporting permanent adoption of the programs. As this project was funded by the ACL, part of the leader training was to educate leaders on the process for requesting participants to complete the data collection forms and on the voluntary nature of participation. The protocol included reading an oral script that served as informed consent at the beginning of each program and then administration of the surveys. Given the limited time and busy schedules of program leaders, over time leaders teaching multiple programs were less likely to follow this request for voluntary participant form completion. Possible solutions include providing incentives (to sites, leaders and/or participants), having RTO staff and graduate students more consistently assist in the data collection, and using technology to improve adherence (e.g., use Google forms instead of paper to improve the process).

1. Develop and nurture a strong community connection.

The NVFPA, from its early days as a steering committee to its current robust membership, served as the communication hub for connecting local community organizations. NVFPA members were critical in identifying interested program leaders and host sites. The quarterly meetings included an education component, project updates and networking. While meetings were held in-person prior to the COVID-19 pandemic, they transitioned to virtual following it, which allowed for greater participation. Members anecdotally reported that the meetings keep them engaged and provided a mechanism to promote falls prevention activities in the community. Project staff maintained a website for the NVFPA, which included detailed listings of current programs, resources for trained leaders and the general public, and links to member websites and regional events. This helped to foster cross-agency collaboration in an effort to increase awareness of falls prevention efforts while minimizing duplication of services.

### Limitations

Limitations discovered through the development of this academic-community partnership can be used to improve similar programmatic efforts as well as focus areas of future work for the project team. Collecting detailed data is a key component of assessment and for this initiative it was limited by the willingness of community partners and leaders to provide data and have surveys completed. Barriers to evaluating implementation models include lack of adherence to data collection (44). Future efforts must include implementing new strategies, including incentives, to improve the readiness of organizations to build data collection into their workflow.

Although EBFPPs continue to be delivered within the region, many unanswered questions remain about program adoption. Analysis of factors associated with sites that have adopted programs from those who have not, as well as what characteristics are associated with maintaining these programs over time is needed. It would also be beneficial to develop a mechanism to capture which trained leaders actually led programs and determine the staff and staff setting variables that separate adopters from non-adopters. Understanding which entities are likely to adopt programs will minimize barriers and frustration, and maximize resources when attempting to deliver EBFPPs.

Delivery of the EBFPPs during the project time period was disrupted by the COVID-19 pandemic. A hiatus in offerings occurred as EBFPP implementation sites stopped all in-person programming. Since older adults were not partaking in programming, data collection halted as well. While not being able to offer in-person classes limited the ability of older adults to participate in beneficial health promotion programs and the project team to meet its goals, it did foster the creation of alternative delivery methods. Virtual programming for MOB and SAIL, a product of the pandemic, continued to be offered in addition to in-person classes, widening opportunities for more older adults to participate in falls prevention activities.

Finally, a desire of the project team was to target older adults at different levels of fall risk. The initiative was successful in reaching community-dwelling older adults with low to moderate risk for falls, but not for those at high risk. OEP was chosen by the project team to provide a program for ambulatory older adults that had a high risk of falling (27, 28). Challenges with reimbursement from Medicare became an obstacle to implementation of the physical therapist-led version of the program, and concerns over liability and participant safety became an issue for local organizations with the lay-leader, community-based format where sites could only provide limited professional oversight. Continued work is needed to identify strategies and supports to implement community-accessible falls prevention programs for individuals at high risk for falls or to make health professional guided programs, such as OEP, more accessible (31, 33).

The location of the project in a large metropolitan area and the implementation strategies employed likely impacted its successes and challenges. Despite these limitations, the overall lessons learned from this community case study provide insights that may encourage and guide others toward delivering multiple EBFPPs in their communities.

## Conclusion

This ACL-funded project had a goal of delivering multiple EBFPPs that have been proven to reduce falls, fear of falling, and fall-related injuries in older adults in a region in which none previously existed. The initial results indicate that using an academic-community partnership model to build relationships and capacity for ongoing delivery of evidence-based programming was successful in achieving this goal. Community-based initiatives such as this one are critically important components of a comprehensive fall prevention strategy.

This academic-community partnership model successfully delivered two EBFPPs in a metropolitan setting, introduced a third, implemented a mechanism to train program leaders, and built a network of invested partners to foster continued support of fall prevention activities and programming. Although all three EBFPPs were made available to all sites, only two sites adopted multiple programs.

Collaborating with community partners is the foundation for this academic-community partnership model. Individuals using it seek to connect research and other academic initiatives with various community members from multiple sectors including public health, health care, for-profit and nonprofit, faith-based and others (45, 46). This work entails being open to partners’ wishes and agendas. Success comes from the collective desire to build capacity to meet community-identified needs.

## Data availability statement

The original contributions presented in the study are included in the article/supplementary material, further inquiries can be directed to the corresponding author.

## Ethics statement

The studies involving human participants were reviewed and approved by Marymount University’s Institutional Review Board. Written informed consent for participation was not required for this study in accordance with the national legislation and the institutional requirements.

## Author contributions

CE led the development and writing of the manuscript. RW provided substantial conceptual input and CE, SP and RW wrote components of it. All authors critically reviewed the manuscript, suggested revisions, and approved the published version.

## Funding

This work was funded by the Department of Health and Human Services, Administration for Community Living, AOA-PPHF Evidence-Based Falls Prevention Programs, Grant #90FPSG0012-01-00 and #90FP0028-01-00.

## Conflict of interest

The authors declare that the research was conducted in the absence of any commercial or financial relationships that could be construed as a potential conflict of interest.

## Publisher’s note

All claims expressed in this article are solely those of the authors and do not necessarily represent those of their affiliated organizations, or those of the publisher, the editors and the reviewers. Any product that may be evaluated in this article, or claim that may be made by its manufacturer, is not guaranteed or endorsed by the publisher.
